# On the Most Favorable Position for Practising Artificial Respiration in Cases of Asphyxia

**Published:** 1856-05

**Authors:** 


*On the most favorable position for practising Artificial Respiration in cases of Asphyxia. (Extract from a Letter of Marshall Hall to M. Flourens.)*

I am at present very much engaged in researches upon asphyxia. I believe I have established the advantage of the prone position when it is desired to practice artificial respiration. If the subject reposes upon the back, the tongue falls upon the epiglottis, which is carried back upon and closes the glottis ; liquids that may be in the mouth or rise into it from the stomach, obstruct the same passage.

Everything, however, is changed by reversing the position and placing the subject upon his face ; the tongue drops forward dragging after it the epiglottis, the glottis opens and the air is allowed to enter freely in inspiration; liquids in the back of the mouth flow out—thus allowing efforts to excite respiration to be more efficacious in the latter position.

I will transmit my labor to you when I shall deem it worthy.—*Nashville Journal, from Gaz. Med. de Paris*, Dec. 8th, 1855.

